# *Bartonella* Spp. in Pets and Effect on Human Health

**DOI:** 10.3201/eid1203.050931

**Published:** 2006-03

**Authors:** Bruno B. Chomel, Henri-Jean Boulouis, Soichi Maruyama, Edward B. Breitschwerdt

**Affiliations:** * University of California School of Veterinary Medicine, Davis, California, USA;; † Microbiologie-Immunologie, Ecole Nationale Vétérinaire d'Alfort, Maisons-Alfort, France;; ‡ Nihon University, Kanagawa, Japan;; § North Carolina State University College of Veterinary Medicine, Raleigh, North Carolina, USA

**Keywords:** *Bartonella*, cat, cat-scratch disease, dog, endocarditis, zoonoses, research

## Abstract

Among the many mammals infected with *Bartonella* spp., pets represent a large reservoir for human infection because most *Bartonella* spp. infecting them are zoonotic. Cats are the main reservoir for *Bartonella henselae*, *B*. *clarridgeiae*, and *B*. *koehlerae*. Dogs can be infected with *B*. *vinsonii* subsp. *berkhoffii*, *B*. *henselae*, *B*. *clarridgeiae*, *B*. *washoensis*, *B*. *elizabethae*, and *B*. *quintana*. The role of dogs as an important reservoir of *Bartonella* spp. is less clear than for cats because domestic dogs are more likely to be accidental hosts, at least in nontropical regions. Nevertheless, dogs are excellent sentinels for human infections because a similar disease spectrum develops in dogs. Transmission of *B*. *henselae* by cat fleas is better understood, although new potential vectors (ticks and biting flies) have been identified. We review current knowledge on the etiologic agents, clinical features, and epidemiologic characteristics of these emerging zoonoses.

## Abstract 2

Pets represent a large reservoir for human infection.

*Bartonella* spp. are fastidious, hemotropic, gram-negative bacteria that are mainly transmitted by vectors. Among the 11 species or subspecies known or suspected to be pathogenic for humans, 6 have been isolated from pet dogs and cats (Table 1). Domestic cats are the principal reservoir for *Bartonella henselae*, the main agent of cat-scratch disease (CSD); *B*. *clarridgeiae*, which has been suspected in a few cases of CSD; and *B*. *koehlerae*, recently reported as the cause of human endocarditis (*1**,**4*). Domestic dogs could be one of the reservoirs for *B*. *vinsonii* subsp. *berkhoffii* (reported as *B*. *v*. *berkhoffii* thereafter) because as it can cause prolonged bacteremia in this species (*5**,**6*). Dogs can also be infected with *B*. *henselae*, *B*. *clarridgeiae*, *B*. *washoensis*, and *B. elizabethae* (*2*). Recently, 2 cases of endocarditis caused by *B. quintana* were diagnosed (P. Kelly et al., unpub. data). As with human disease, the clinical spectrum of *Bartonella* infection in dogs is expanding (*2*). Fleas play a major role in the transmission of feline *Bartonella* (*7*), but other potential vectors, such as ticks and biting flies have been recently identified to harbor *Bartonella* DNA, including *B. henselae* (*8**,**9*). This article provides an update on the etiologic agents, new clinical features, and evolving epidemiologic characteristics of these emerging zoonoses. We will not discuss the diagnosis, treatment, and prevention of *Bartonella* infections, as several recent review articles have been written on this subject (*1**,**2**,**10*).

**Table 1:** Species and subspecies of Bartonella that are confirmed or potential human pathogens

*Bartonella* sp.	Primary reservoir	Vector	Accidental host	Reference
*B*. *bacilliformis*	Human	Sandfly (*Lutzomia verrucarum*)	None	(*1**,**2*)
*B*. *quintana*	Human	Body louse (*Pediculus humanis*)	Cat, dog, monkey	(1–3, P. Kelly et al., unpub. data)*
*B*. *elizabethae*	Rat (*Rattus norvegicus*)	Oriental rat flea (*Xenopsylla cheopis*)	Human, dog	(*2*)
*B*. *grahamii*	Wild mice (*Clethrionomys glareolus*, *Microtus agrestis*, *Apodemus flavicollis*)	Rodent fleas	Human	(*1**,**2*)
*B*. *henselae*	Cat (*Felis catus*)	Cat flea (*Ctenocephalides felis*)	Human, dog	(*1**,**2*)
*B*. *clarridgeiae*	Cat	Cat flea	Human?, dog	(*1**,**2*)
*B*. *koehlerae*	Cat	Cat flea	Human	(*2**,**4*)
*B*. *vinsonii berkhoffii*	Coyote (*Canis latrans*), dog (*C*. *familiaris*)	Unknown (ticks?)	Human	(*5**,**6*)
*B*. *vinsonii arupensis*	White-footed mouse (*Peromyscus leucopus*)	Unknown (fleas?, ticks?)	Human	(*1**,**2*)
*B*. *washoensis*	California ground squirrel (*Spermophilus beecheyii*)	Unknown (fleas?)	Human, dog	(*2*)
*B*. *alsatica*	Rabbit	Unknown (flea?)	Human	(D. Raoult, pers. comm.)

*Also reported by O'Rourke LG, Pitulle C, Hegarty BC, Kraycirik S, Killary KA, Grosenstein P, et al. *Bartonella quintana* in cynomolgus monkey (*Macaca fascicularis*). Emerg Infect Dis. 2005;11:1931-4.

## Feline *Bartonella* Species

### *B*. *henselae*

Since the first isolation of *B*. *henselae* from a domestic cat in the early 1990s, several studies have been conducted worldwide to determine the importance of cats as a reservoir of this bacterium (reviewed in [2]). Prevalence of infection varies considerably among cat populations (strays or pets) with an increasing gradient from low in cold climates (0% in Norway) to high in warm and humid climates (68% in the Philippines) (*2*). At least 2 genotypes have been identified and designated Houston-1 (type I) and Marseille (previously BATF) (type II) (*1**,**2*). The respective prevalence of these 2 genotypes varies considerably among cat populations from different areas. *B. henselae* type Marseille is the dominant type in cat populations in the western United States, western Europe (France, Germany, Italy, the Netherlands, United Kingdom), and Australia, whereas type Houston-1 is dominant in Asia (Japan and the Philippines) (reviewed in [2]). However, within a given country, the prevalence may also vary among cat populations. For instance, in France, Marseille type was the most common type in cats from the Nancy and Paris areas, whereas type Houston-1 was the main genotype in cats from Lyon or Marseille (references cited in [2]). However, a few studies in western Europe and Australia have reported that most human cases of CSD were caused by *B. henselae* type Houston-1, despite the fact that type Marseille was found to be the dominant type in the cat population, which suggests that type Houston-1 strains could be more virulent to humans (*2*). Cats are usually bacteremic for weeks to months, but some cats have been reported to be bacteremic for >1 year. Young cats (<1 year) are more likely than older cats to be bacteremic (*11*), and stray cats are more likely to be bacteremic than pet cats (*1**,**2*).

The clinical description of CSD was first reported in France by Debré et al. in 1950, but the etiologic agent was identified only in 1992 (*1**,**2**,**6*). The annual number of cases in the United States has been estimated to be between 22,000 and 24,000, with ≈2,000 cases that require hospitalization, and thousands of cases may occur yearly in Europe. In various studies, the seroprevalence of antibodies to *B*. *henselae* in healthy persons has ranged from 3.6% to 6% (Table 2) and could be higher in some specific population groups, such as veterinarians, children, or elite orienteers (orienteering is a sport in which participants compete to find points in the landscape using a map and compass). Table 2 gives comparative *B*. *henselae* seroprevalence data for cat and healthy human populations from selected countries, which suggests that seroprevalence is low in both cats and humans at northern latitudes and increases in warmer climates (*11**–**24*). Such data are informative and cannot exclude possible serologic cross-reactivity with some other *Bartonella* spp.

**Table 2:** *Bartonella henselae* seroprevalence in various cat and human populations from selected countries*

Country	Cat seroprevalence (%)			Human seroprevalance (%)		
	Stray	Pet	Reference	Healthy	Other	Reference
Sweden	NA	1	(*19*)	1	NA	(*12*)
Japan	NA	8.8–15.1; northern, 0–2; central 10.9–12.6; southern, 18–24	(*20*)	4.5	11.0–15.0 (veterinarians)	(*13**,**14*)
United States	81	27.9	(*11**,**21*)	3.6–6	7.0 (veterinarians)	(*15*)
Thailand	27.6†	NA	(*22*)	5.5	NA	(*16*)
Italy	39.0	43.5	(*23*)	NA	8.5–61.6 (children)	(*17*)
Jordan	NA	32.0	(*24*)	NA	NA	(*18*)

*NA, not available. †Prevalence of bacteremic cats; no data available on seroprevalence.

Despite the fact that *B*. *henselae* infection can cause meningitis and encephalitis, only 1 case of a fatal infection has been reported (*5*). CSD is more frequently observed in persons <20 years of age and in persons who own a young cat (<1 year of age, especially if this cat is infested with fleas) or in persons who have been scratched or bitten by a cat (*1**,**2**,**6*). In immunocompetent persons, CSD is mainly characterized by a benign regional lymphadenopathy. Usually after a cat scratch, a papule and then a pustule develop within 7 to 12 days at the injection site, followed by a regional lymphadenopathy (usually involving a single lymph node) 1–3 weeks later that can persist for few weeks to several months. Low-grade fever, malaise, and aching are often reported; in some instances, headache, anorexia, and splenomegaly can occur. Abscessed lymph nodes are reported occasionally. In 5% to 9% of CSD patients, atypical manifestations may develop, including Parinaud oculoglandular syndrome, encephalitis, endocarditis, hemolytic anemia, hepatosplenomegaly, glomerulonephritis, pneumonia, relapsing bacteremia, and osteomyelitis.

On the basis of serologic testing or polymerase chain reaction (PCR), several recent publications have associated *B. henselae* with uveitis, focal retinal phlebitis, neuroretinitis, retinal and optical nerve neovascularization, and retinal artery and vein occlusions. Neurologic forms are rare, and patients usually completely recover within 1 year without sequelae. Hepatosplenomegaly and osteolytic bone lesions have been described in persons seropositive for *B. henselae*. Pseudotumoral lesions involving the mammary glands, the liver, or the spleen and, recently, glomerulonephritis and cases of monoclonal and biclonal gammopathy have also been associated with *B*. *henselae* antibodies. Cases of prolonged fever without adenopathy, chronic fatigue, hemolytic anemia, thrombocytopenic purpura, Henoch-Schönlein purpura syndrome, pleuritis, pneumonia, and even paronychia have been reported in patients who were seropositive for *B*. *henselae* (*1**,**2*). Usually, these clinical manifestations disappear in a few weeks to a few months. Bacteremia is rarely detected in immunocompetent persons. Several cases of endocarditis have been associated with *B*. *henselae* infection, most frequently in persons with preexisting valvular lesions. Besides *B*. *henselae*, most human cases of *Bartonella* endocarditis are caused by *B*. *quintana*, but a few cases of endocarditis or myocarditis have been associated with *B*. *elizabethae* (1 case), *B*. *vinsonii berkhoffii* (1 case), *B*. *vinsonii arupensis* (1 case), *B*. *koehlerae* (1 case), *B*. *washoensis* (1 case), and *B*. *alsatica* (1 case) (Table 3).

**Table 3:** Clinical aspects of *Bartonella* infections in humans and dogs

*Bartonella* sp.	Symptoms	
	Humans	Dogs
*B*. *clarridgeiae*	Cat-scratch disease	Endocarditis, lymphocytic hepatitis
*B*. *elizabethae*	Endocarditis, neuroretinitis	Lethargy, anemia, weight loss
*B*. *henselae*	Cat-scratch disease, endocarditis, bacillary angiomatosis, (peliosis hepatis), granulomatous hepatitis, pseudotumoral lesions, arthritis, arthralgia, osteomyelitis, nodules, erythema, cutaneous petechiae, uveitis, neuroretinitis, purpura (Henoch-Schönlein), glomerulonephritis, perionyxis, periodontitis	Granulomatous hepatitis, peliosis hepatis, epistaxis
*B*. *grahamii*	Neuroretinitis, bilateral retinal artery branch occlusions	Not diagnosed in dogs
*B*. *koehlerae*	Endocarditis	Not diagnosed in dogs
*B*. *vinsonii* subsp. *arupensis*	Bacteremia, fever, arthralgia, neurologic disorders, endocarditis	Not diagnosed in dogs
*B*. *vinsonii* subsp. *berkhoffii*	Endocarditis	Endocarditis, myocarditis, arrhythmia, uveitis, choroiditis, limping, splenomegaly, polyarthritis, epistaxis
*B*. *washoensis*	Fever, myocarditis	Endocarditis
*B*. *quintana*	Fever, bacteremia, endocarditis, bacillary angiomatosis	Endocarditis

In immunocompromised patients, *B*. *henselae* infection can cause prolonged fever, prolonged bacteremia, or both (*1**,**2**,**6*). Bacillary angiomatosis or peliosis is usually observed in highly immunocompromised persons (low CD4 count), who often are infected with HIV. Several severe infections have also been reported in organ transplant recipients (*1**,**2*).

The clinical spectrum of the infection in cats has not been fully investigated, but naturally infected cats primarily seem to be healthy carriers of the bacterium (*1**,**2**,**6*). However, cases of uveitis and rare cases of endocarditis have been molecularly associated with infection caused by *B*. *henselae*. Seropositive cats were more likely to have kidney disease and urinary tract infections, stomatitis, and lymphadenopathy. In experimentally infected cats, fever, lymphadenopathy, mild neurologic signs, and reproductive disorders have been reported.

### *B*. *clarridgeiae*

*B*. *clarridgeiae* was first isolated in the United States from the pet cat of an HIV-positive patient (*25*). This *Bartonella* sp. has been less frequently isolated from domestic cats than *B*. *henselae* because it appears to be more difficult to isolate and is unevenly distributed in cat populations worldwide. A *B*. *clarridgeiae* prevalence of 17% to 36% among all *Bartonella* isolates was reported in studies conducted in France, the Netherlands, the Philippines, and Thailand (*2**,**22*). However, *B*. *clarridgeiae* represented <10% of all isolates from domestic cats in the southeastern United States, Japan, or Taiwan (*2*) and has never been isolated in studies conducted in Europe, Australia, and North America (*2*). No specific pathologic features have been associated with natural infection in cats. However, in experimentally coinfected cats (*B*. *henselae* type II and *B*. *clarridgeiae*), clinical signs were minimal, and gross necropsy results were unremarkable, but histopathologic examination showed peripheral lymph node hyperplasia, splenic follicular hyperplasia, lymphocytic cholangitis/pericholangitis, lymphocytic hepatitis, lymphoplasmacytic myocarditis, and interstitial lymphocytic nephritis (*26*). In humans, *B*. *clarridgeiae* has never been isolated or detected by molecular methods. However, *B*. *clarridgeiae* could be a minor causative agent of CSD, as the presence of *B*. *clarridgeiae* antibodies were reported in a suspect case of CSD and in a patient with a chest-wall abscess (reviewed in [2]). Furthermore, anti-flagella (FlaA)–specific antibodies against *B*. *clarridgeiae* were detected by immunoblotting in 28 (3.9%) of 724 patients with lymphadenopathy but in none of 100 healthy controls. However, substantial cross-reactivity between *B*. *henselae* and *B*. *clarridgeiae* detected by indirect fluorescence antibody assay was noted in human sera in a recent study from Japan (*2*).

### B. koehlerae

*B*. *koehlerae* is a *Bartonella* sp. that has rarely been isolated from domestic cats worldwide, as it is a very fastidious bacterium (*2**,**4*). Until recently, it had been isolated only from 2 cats in California and 1 cat in France (*2**,**4**,**27*). The first human case of *B*. *koehlerae* endocarditis was reported from Israel in 2004 (*2*). Furthermore, these authors were able to isolate *B*. *koehlerae* from a bacteremic stray cat from that country.

### *B*. *quintana* and *B*. *bovis*

A few suspect cases of CSD and cases of bacillary angiomatosis or endocarditis have been associated with *B*. *quintana*, for which the only risk factor identified was a contact with cats or cat fleas (*3*). Furthermore, the identification of *B*. *quintana* DNA in cat fleas (*28*) and recently in the dental pulp of a cat (*3*) has raised the question as to whether cats might be a possible source of human infection. However, *B*. *quintana* has not yet been isolated from naturally infected cats anywhere in the world where epidemiologic studies have been conducted to detect *Bartonella*-bacteremic cats. Similarly, 2 cats infected with *B. quintana* did not become bacteremic but seroconverted (*29*). Subsequently, both cats became bacteremic when challenged with *B*. *henselae*.

A few cases of *B*. *bovis* (formerly *B*. *weissii*) infections have been reported in cats from Illinois and Utah in the United States (*1*). The epidemiologic role of cats for this organism is still unknown.

## Dogs as Sentinels for Human Infections?

Dogs can be infected with *B*. *v*. *berkhoffii*, *B*. *henselae*, *B*. *clarridgeiae*, *B*. *washoensis*, *B*. *elizabethae*, and *B*. *quintana* (2, P. Kelly et al., unpub. data). However, the role of dogs as a major reservoir of *Bartonella* spp. is not clear. Current evidence suggests that domestic dogs are more likely to be accidental hosts of various *Bartonella* spp., at least in nontropical regions. Nevertheless, domestic dogs could be one of the reservoirs for *B*. *v*. *berkhoffii*, as it causes prolonged bacteremia in this species (*5**,**6*). The epidemiologic situation is quite distinct between tropical areas where several studies have shown a high prevalence of *B*. *v*. *berkhoffii* antibodies, especially in stray dogs, and more northern latitudes, where very low antibody prevalence has been detected in domestic dogs, especially among pets. In sub-Saharan Africa, seroprevalence of 26% in dogs in Senegal and up to 65% in native dogs from Sudan has been reported (*1*). In North Africa, we found that 38% of 147 dogs from Morocco were seropositive for *B*. *v*. *berkhoffii* (*30*). In 113 dogs from the Reunion Island, in the Indian Ocean, a seroprevalence of 18% was reported in stray dogs, whereas only 3% of dogs examined at veterinary clinics were seropositive, and no dog was bacteremic (*31*). In Thailand, 38% of sick dogs who exhibited fever, anemia, or thrombocytopenia were seropositive for *B*. *v*. *berkhoffii* (*1*). On the contrary, studies in the United States and Europe reported a seroprevalence of <5% in domestic dogs; selected dog populations were at higher risk, including rural dogs and government working dogs (*2*). However, concerns about false-positive results in animals should be raised, as specificity and sensitivity of the tests for dogs have not been fully evaluated. In California, *B*. *v*. *berkhoffii* has rarely been isolated from domestic dogs or detected by PCR, whereas coyotes (*Canis latrans*) appear to be a reservoir of this pathogen, as 35% of the coyotes tested in California were seropositive, and 28% of the coyotes tested within a highly disease-endemic region of California were bacteremic (*2*).

In domestic dogs, *B*. *v*. *berkhoffii* is a cause of endocarditis (*6*) and, as in humans, the clinical spectrum of the infection attributed to this organism is expanding. *B*. *v*. *berkhoffii* is now associated with cardiac arrhythmias, endocarditis and myocarditis, granulomatous lymphadenitis, granulomatous rhinitis, and epistaxis (*6**,**32*). In both humans and dogs, *Bartonella*-associated cases of endocarditis usually involve the aortic valve and are characterized by massive vegetative lesions (*33*). Based on serologic evidence, infection with *B*. *v*. *berkhoffii* may also cause immune-mediated hemolytic anemia, neutrophilic or granulomatous meningoencephalitis, neutrophilic polyarthritis, cutaneous vasculitis, and uveitis in dogs (*2*).

Some other *Bartonella* spp. have infrequently been isolated from domestic dogs. *B*. *clarridgeiae* and *B*. *washoensis* were isolated from cases of endocarditis (*1**,**2*), and *B*. *henselae* was isolated for the first time from a dog from Gabon (*34*). In the Gabon study, *B*. *clarridgeiae* was isolated from 5 of 258 dogs tested (1.9%), which suggests a possible reservoir role for this *Bartonella* sp. in Africa (*34*). *B. henselae*, *B. elizabethae*, and *B*. *clarridgeiae* DNA has also been detected from a few sick dogs with various clinical abnormalities (Table 3) (*1**,**2**,**6*). Endocarditis caused by *B. quintana* was recently diagnosed in a dog from the United States and a dog from New Zealand (P. Kelly et al., unpub. data). Two recent studies reported a *B*. *henselae* antibody prevalence of 10% in healthy dogs in the eastern United States (*35*) and a prevalence of 14% of dogs in Zimbabwe (*36*). A much higher prevalence (27%) in sick dogs from the eastern United States was reported (*35*), which contrasts with the low *B*. *henselae* seroprevalence (<2%) in dogs examined at a university teaching hospital in northern California (*37*). A case-control study conducted on 305 dogs (102 dogs seropositive for *B*. *henselae*, *B*. *v*. *berkhoffii*, or *B*. *clarridgeiae* and 203 seronegative dogs) suggested an association between the seropositive status and lameness, arthritis-related lameness, splenomegaly, and nasal discharge/epistaxis (*37*).

Unlike the domestic cat, for which clinical manifestations of natural infection is rarely documented, a wide range of clinical and pathologic abnormalities develop in dogs that are very similar to those observed in humans (*32*). Therefore, this species is an excellent sentinel and an important comparative model for human infections. To date, all *Bartonella* spp. identified in sick dogs are also pathogenic or potentially pathogenic in humans.

## Beyond the Fleas: New Emerging Vectors

The primary mode of transmission of *B. henselae* to humans is through a cutaneous trauma caused mainly by the scratch of a cat. Transmission is less likely to occur by cat bite; shedding of *B*. *henselae* in cat saliva has not been clearly documented. The possibility of direct transmission of *B*. *henselae* to humans by the cat flea is something that has not been proven experimentally and is mainly hypothetical. However, the presence of cat fleas (*Ctenocephalides felis*) is essential for the maintenance of the infection within the cat population (*6*). *B*. *henselae* has been shown to multiply in the digestive system of the cat flea and survive several days in the flea feces (reviewed in [2]). Experimentally, only cats inoculated with flea feces compared to those on which fleas were deposited in retention boxes or that were fed fleas became bacteremic (*38*). Therefore, the main source of infection appears to be flea feces that are infected by contaminated cat claws.

Beside the cat flea, new possible vectors have been suggested. *Bartonella* DNA, including *B*. *henselae*, has been detected in *Ixodes ricinus* ticks collected on humans (*9*) and in *I. scapularis* ticks collected in households of persons coinfected with *B. henselae* and *Borrelia burgdorferi* (reviewed in [2]). *B*. *quintana*, *B*. *henselae*, and *B*. *v*. *berkhoffii* DNA were also detected in questing *I*. *pacificus* ticks in California, and a few human cases of *B*. *henselae* infection were temporally related to a tick exposure in the United States (reviewed in [2]). Tick exposure was reported as a risk factor associated with CSD in humans (*39*). Similarly, tick exposure was determined to be a risk factor associated with *B*. *v*. *berkhoffii* seropositivity in dogs (*40*). Additional indirect support for ticks as vectors of *B*. *v*. *berkhoffii* in dogs relates to serologic or PCR evidence of concurrent infections with various tickborne organisms (*6**,**33*). The specific role of ticks in *Bartonella* transmission requires additional study, but several recent publications have reported a high prevalence of *Bartonella* spp. infection in ticks from various parts of the world. Finally, *B*. *henselae* type Marseille DNA was recently detected in a stable fly (*8*).

## Conclusion

The number of zoonotic *Bartonella* species identified in the last 15 years has increased considerably. Pets have been identified as a notable reservoir of *Bartonella* species (i.e., cats and *B*. *henselae* or dogs and *B*. *v*. subsp. *berkhoffii* in the tropics) and may play an important role as source for human infection. Furthermore, domestic dogs may represent excellent sentinels for *Bartonella* infection because of the wide diversity of the *Bartonella* spp. identified in canines, all of which are human pathogens. A better understanding of the modes of transmission and vectors involved in dog bartonellosis is an urgent priority to implement appropriate parasite control measures for pets.
